# First Application of QuEChERS-GC-MS Analysis for Polycyclic Aromatic Hydrocarbons Detection in Human Adipose Tissue

**DOI:** 10.1021/acsomega.5c06719

**Published:** 2026-02-25

**Authors:** Alice Franchin, Elena Gregoris, Luca Sorarù, Elena Stocco, Vincenzo Vindigni, Andrea Porzionato, Daniele Brunelli, Veronica Macchi, Andrea Gambaro, Marco Roman

**Affiliations:** † 19047 Ca’ Foscari University of Venice, Department of Environmental Sciences, Informatic and Statistics, via Torino 155, Venice (Mestre), Veneto 30172, Italy; ‡ National Research Council, Institute of Polar Sciences, via Torino 155, Venice (Mestre), Veneto 30172, Italy; § 9308 University of Padua, Department of Neurosciences, via Belzoni 160, Padova, Veneto 35121, Italy

## Abstract

Biomonitoring plays a crucial role in assessing human exposure to hazardous substances by determining the presence and concentration of pollutants in the body. This study is part of the PRIN 2022 PNRR project “Integrated systemic detection of pollutants in the human body” (INSYDE-HU), which focuses on developing analytical methods to quantify selected pollutants in human tissues from nonoccupationally exposed individuals. The aim of this research is to develop and validate a reliable analytical methodology for the quantitative determination of toxic polycyclic aromatic hydrocarbons (PAHs) in human adipose tissue, a complex and rarely studied matrix in biomonitoring due to the invasiveness of sampling. PAHs are lipophilic, toxic compounds widely present in the environment and are prone to bioaccumulate in fat tissue. The method was developed by using the QuEChERS extraction technique followed by analysis through gas chromatography coupled with mass spectrometry (GC-MS). Validation was performed on bovine fat, followed by application to human adipose tissue samples provided by the Plastic Surgery Unit of the University Hospital of Padua. Fourteen PAHs were validated with method detection (MDLs) and quantification (MQLs) limits ranging from 0.11 to 4.13 and 0.30 to 13.76 ng g^–1^, respectively. Phenanthrene, anthracene, and pyrene were detected in human samples, with pyrene quantified between 2.34 and 4.88 ng g^–1^. To our knowledge, this is one of the few methods for the determination of these compounds in such a complex matrix and the only one that successfully combines QuEChERS and GC-MS.

## Graphical abstract

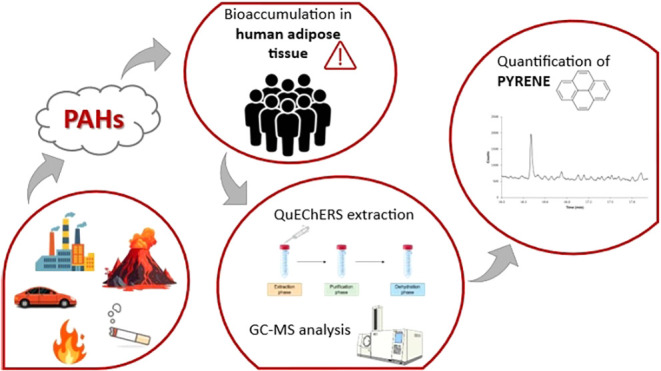

## 1 Introduction

Polycyclic aromatic hydrocarbons (PAHs) are a group of ubiquitous, toxic, mutagenic, and carcinogenic compounds classified by the European Commission as priority pollutants.^1^ Human exposure to PAHs is continuous and widespread due to their environmental ubiquity, occurring through the ingestion of contaminated food, inhalation of polluted air, and dermal exposure. Among these, dietary intake is the primary route of exposure. In fact, food contamination by PAHs can result from both environmental pollution and food preparation and processing (particularly during smoking or frying processes).^2,3^ Because of their lipophilicity, PAHs preferentially accumulate in fatty tissues, raising concerns about long-term storage and possible links to cancer, obesity, and cardiovascular diseases.^2–4^ While animal studies have demonstrated accumulation in porcine, bovine,^5^ rodents,^6^ fish,^7,8^ and mussle^9,10^ adipose tissue, only limited data are available for humans. The lack of reference methods for human adipose tissue analysis highlights a critical gap in biomonitoring research. For that reason, it is crucial to implement biomonitoring measures for the removal of toxic pollutants.

Adipose tissue is a highly relevant, yet challenging matrix for analysis due to its high lipid content, which can interfere with extraction and instrumental analysis, reducing recovery and reproducibility.^11^ Furthermore, sample collection requires invasive medical procedures and complex sample processing.^12^ For this reason, previous studies often focused on less invasive matrices such as urine, blood, or serum.^13–16^ However, these provide only indirect information about long-term accumulation. Analysis of adipose tissue remains essential as it represents the most relevant storage site for lipophilic pollutants.^12^

Traditional procedures for PAH analysis in complex matrices involve time-consuming multistep approaches, such as Soxhlet extraction, solid phase extraction (SPE), and accelerated solvent extraction (ASE),^17^ often coupled with gas chromatography–mass spectrometry (GC-MS), due to its high selectivity and sensitivity. For example, previous studies on human adipose tissues used UAE and analysis by HPLC^12^ salt-assisted liquid–liquid extraction (SALLE), followed by a dispersive liquid–liquid microextraction (DLLME) and GC-MS analysis^18^ or Soxhlet extraction followed by a GC-MS analysis.^19^ While effective, these extraction techniques are typically very time-consuming and use large quantities of solvents.

In contrast, the QuEChERS method (which stands for Quick, Easy, Cheap, Effective, Rugged, and Safe) could be a valid approach that offers a rapid and cost-effective alternative, with reduced sample and solvent use^20,21^ and high applicability to complex matrices. Developed initially by Anastassiades et al.^22^ to determine the presence of pesticides in plant-based foods, the QuEChERS method has shown high performance in the analysis of PAHs in animal tissues: recently, Kiełbasa and Buszewski^23^ demonstrated higher recovery values of QuEChERS (from 84 to 101%) compared with UAE (from 54 to 68%) to investigate the presence of PAHs in porcine, avian, cod, and herring tissues. Furthermore, the QuEChERS method was estimated to reduce the analysis time from hours to about 20 min and save the extraction costs of more than 60% with respect to the use of SPE for environmental solids.^24^

Despite the demonstrated efficiency of the QuEChERS extraction method and the robustness of the GC-MS analysis, no previous study combined these two approaches for the determination of PAHs in human adipose tissues. Given the importance of investigating human exposure to environmental pollutants and the significant impact of PAHs, especially the high-molecular weight congeners,^25^ on human health, a need for a highly efficient and standardized analytical method is critical. The combination of QuEChERS with GC/MS could represent an optimal option for the challenging adipose tissue matrix, and thus, the present work aims at developing and validating an analytical method for the quantitative determination of PAHs in human adipose tissue using QuEChERS extraction followed by GC-single quadrupole MS analysis. Preliminarily, a preanalytical phase was developed and optimized using bovine adipose tissue as a surrogate matrix and then applied to real human samples. The use of animal tissues or synthetic matrices as a surrogate for human tissue is a well-established practice in the literature, particularly when human specimens are scarce or difficult to procure.^26–31^ To the best of our knowledge, this represents the first study to implement this combined approach for human adipose tissue biomonitoring.

## 2 Experimental Section

### 2.1 Sampling

Due to the limited availability and the invasive nature of collecting human adipose tissue, bovine adipose tissue was employed as a surrogate matrix for method validation and construction of the matrix-match calibration curve, following the principle of surrogate matrix use in bioanalytical protocols.^26^ This choice is justified by the high degree of biochemical similarity between the two tissues: both are composed of over 90% of triglycerides, with the fatty acid profiles of both species dominated by linear long-chain fatty acids, primarily palmitic (C16:0) and oleic (C18:1) acids, which together account for the majority of the lipid mass.^31,32^ Consequently, this approach allowed us to reserve the entire set of available human specimens for actual sample analysis, avoiding the consumption of precious clinical material during the preanalytical optimization.

Bovine adipose tissue was obtained from a local butcher’s shop and then kept at −20 °C until use. Human adipose tissue was provided by the Operative Complex Unit of Plastic Surgery of the University Hospital of Padua after receiving institutional review board approval (CESC Code 4502/AO/2018). Informed written consent was also obtained from all of the participants. Adipose tissue samples were harvested from postbariatric patients with a BMI ≤ 30, a clinical criterion used to include them in the Plastic Surgery Program. A total of 10 samples were isolated from female patients (*n* = 10) who underwent abdominoplasty, liposuction, prosthesis removal, and mastopexy. Specifically, 7 specimens were collected from the abdomen, 2 from the arms, and 1 from the breast. To limit contamination, sample manipulation was reduced to the minimum necessary. Samples were collected into plastic sterile vials and immediately stored at −80 °C. The cold chain was meticulously maintained during transportation from the university hospital to the analysis laboratory.

Within the laboratory, before extraction, all the samples were ground with liquid nitrogen, homogenized using a mortar and pestle, and subdivided, when possible, into two aliquots.

Table 1 lists the analyzed human samples, the sampling area, the age of the patients, and the weight of each aliquot.

**Table 1:** Sample Code, Body Area, and Sample Aliquots

**sample number**	**body area**	**age y**	**aliquot n.**1 g	**aliquot n.**2 g
**#1**	abdomen	42	1.156	1.234
**#2**	arm	27	1.038	0.888
**#3**	abdomen	49	1.143	1.062
**#4**	abdomen	54	1.066	
**#5**	arm	64	1.102	0.873
**#6**	breast	68	1.172	
**#7**	abdomen	51	1.002	1.022
**#8**	abdomen	47	1.133	1.015
**#9**	abdomen	43	1.190	1.076
**#10**	abdomen	40	1.147	1.112

### 2.2 Standards, Reagents, and Materials

The stock solutions of standards used for quantification are PAH-Mix 9 of 10 μg mL^–1^ in acetonitrile containing naphthalene (NA), acenaphthylene (ACL), acenaphthene (AC), fluorene (FL), phenanthrene (PHE), anthracene (AN), fluoranthene (FA), pyrene (Y), benzo­[*a*]­anthracene (BaA), chrysene (CHR), benzo­[*b*]­fluoranthene (BbF), benzo­[*k*]­fluoranthene (BkF), benzo­[*a*]­pyrene (BaP), benzo­[*g,*h*,i*]­perylene (BgP), dibenzo­[*a,h*]­anthracene [DhP] produced by LGC Dr Ehrenstorfer, ^13^C_6_-ACL (100 μg mL^–1^, Cambridge Isotope Laboratories, cod CLM-2477–1.2), ^13^C_6_–PHE (100 μg mL^–1^, Cambridge Isotope Laboratories, cod. CLM-2451–1.2), and ^13^C_4_–BaP (100 μg mL^–1^, Cambridge Isotope Laboratories, cod. CLM-2722–1.2).

The stock solutions were used to prepare intermediate working solutions of native and internal standards of 1 μg mL^–1^.

As solvents were used: acetonitrile (ACN) HiPerSolv (HPLC-MS grade) VWR Chemicals, ethyl acetate (EtAc) anhydrous 99.9% Sigma-Aldrich, 2,2,4-trimethylpentane ROMIL-SpS SuperPurity, ultrapure water (18.2 MΩ·cm, TOC < 20 ppb), obtained using the PURELAB Pulse system ELGA Labwater, *n*-hexane SuperPurity Solvent (Romil), dichloromethane stabilized with amylene SuperPurity Solvent (Romil).

For the QuEChERS method, the following extraction and purification salts were used: MgSO_4_ (VWR Life Science), NaCl (Sigma-Aldrich), sodium acetate (NaAc, VWR Life Science), Agilent Bond Elut Enhanced Matrix Removal-Lipid (EMR-Lipid) purification salt, Agilent AOAC purification salt (composed of MgSO_4_, Primary-Secondary Amine PSA and C_18_EC), and Supel Que Z-Sep purification salt (code 55403-U).

### 2.3 Decontamination and Laboratory Practice Procedure

To reduce contamination levels, all materials and equipment in contact with the samples underwent rigorous cleaning and decontamination procedures. Glassware was precleaned with laboratory-grade detergent and muffled at 400 °C for 4 h. Plasticware and other nonmuffleable components were rinsed with organic solvents (e.g., acetonitrile) and air-dried under a fume hood. Dehydration salts used for the QuEChERS extraction were previously heated in an oven at 150 °C for 24 h, followed by three washes with dichloromethane and *n*-hexane in an ultrasonic bath (each for 10 min) and stored under hexane to prevent rehydration. For the analysis of real samples, glass centrifuge tubes were preferred over plastic materials to minimize the risk of contamination.

After the use, all materials in contact with the biological samples were sterilized in an autoclave (121 °C) and then soaked in water and liquid detergent for at least 24 h, before cleaning and washing. Before storing the material for subsequent use, the muffleable material was muffled at 400 °C for at least 4 h.

### 2.4 Exploratory Tests for the Determination of PAHs

For the optimization of the QuEChERS method, this study tested a series of variables reported in the literature,^7,8,33,40^ specifically three different options for each of the following four parameters: (i) type of solvent (ACN/EtAc/Mix, consisting in a mixture of ACN, EtAc and 2,2,4-trimethylpentane in 2:2:1 *v/v* ratio); (ii) presence and type of extraction salts (no one/ex-AOAC (composed of 0.8 g MgSO_4_ and 0.2 g of NaAc)/standard (consists of MgSO_4_ and NaCl in a 4:1 *w/w* ratio)); (iii) purification substrate (lipid/Z-Sep/pur-AOAC); (iv) amount of purifying agent (0.075/0.225/0.450 g). The tests aim to compare the performance of the possible procedures, particularly to evaluate their ability to produce accurate results.

Since no certified reference standards are commercially available, fortified samples were used in this study. In particular, a known amount of native standard (50 ng for all analytes) is added and then requantified afterward.

To optimize the tests to be carried out, the Taguchi experimental design method was applied.^34^ The design applied in this study is P4L3 (also called L9), which corresponds to testing four parameters (P) at three levels (L) by performing only 9 tests instead of the 81 (L^P^, in this case 3^4^) required by the full-factorial design. Further details about the application of the Taguchi method in the development of bioanalytical methods, and the original Taguchi tables are available in the SOP 04 produced within the INSYDE-HU project.^35^ The characteristics of the tests are shown in Table 2.

**Table 2:** Exploratory Tests Optimized Using the Taguchi Method, P4L3 Design

**# test**	**solvent**	**extraction salt**	**purification salt**	**quantity of purification salt**(g)
**T1**	ACN	no one	lipid	0.450
**T2**	ACN	ex-AOAC	Z-Sep	0.075
**T3**	ACN	standard	pur-AOAC	0.225
**T4**	EtAc	no one	Z-Sep	0.225
**T5**	EtAc	ex-AOAC	pur-AOAC	0.450
**T6**	EtAc	standard	lipid	0.075
**T7**	mix	no one	pur-AOAC	0.075
**T8**	mix	ex-AOAC	lipid	0.225
**T9**	mix	standard	Z-Sep	0.450

In the case of the experimental procedure that involves the use of extraction salts (T2, T3, T5, T6, T8, T9), the following steps were carried out: 1 g of the extraction salts and 1 g of the bovine fat sample fortified with 50 ng of native standard were placed in a centrifuge tube. 50 ng of labeled standard, 5 mL of solvent, and 2 mL of milli-Q water were added. The tubes were shaken with an Orbital Shaker IKA-VIBRAX-VXR at 400 rpm for 3 min and then centrifuged with a Centrifuge OHAUS FRONTIER 5916 for 5 min at 10,000 rpm (14,530 RCF). The supernatant was collected and transferred to the purification phase tubes, which were shaken at 400 rpm for 3 min and then centrifuged for 3 min at 10,000 rpm (14,530 RCF). The supernatant was transferred to the GC vials and analyzed.

In the conventional protocols, water is added to enhance extraction efficiency and recovery of pollutants by optimizing solvent interaction and facilitating the salting-out process.^36–39^ However, some studies report that adding water decreases the solubility of PAHs and negatively affects absolute recoveries.^7,39^ For this reason, in the absence of extraction salts (T1, T4, and T7), we avoided adding extra water. However, a final dehydration step was performed, as reported in Lucas et al.^7^ by using MgSO_4_ and NaCl in a 4:1 w/w ratio as dehydrated salts, and applying the same experimental conditions of the purification step (3 min of agitation at 400 rpm, 5 min of centrifugation at 14,530 RFC).

### 2.5 Confirmation Tests

The tests carried out following the Taguchi method allow for a preliminary selection of the hypothetically best operating conditions for sample preparation.^41^ However, it is advisable to perform additional confirmation tests and assessments on small variations in the experimental conditions to verify the optimal conditions. Based on the results obtained, further tests were conducted, selecting the best options that emerged from the previous experiments (3.1.2. Confirmation tests).

In addition to the traditional QuEChERS sample preparation phases, the possibility of performing a final concentration step under a N_2_ stream was evaluated, concentrating the supernatant to a volume of 150–200 μL. Concentrating the extract can enhance the analyte signal by injecting a more concentrated solution into the GC.

### 2.6 GC-MS Analysis and Method Validation

For the analysis and quantification of PAHs present in the samples, a gas chromatograph (Agilent Technologies 7890A GC System) coupled with a single quadrupole mass spectrometer (Agilent Technologies 5975C VL MSD) was used applying a chromatographic method specifically developed for this purpose. Briefly, the GC was equipped with a split/splitless injector operating in splitless mode at a temperature of 300 °C. The helium flow rate was set to 1.2 mL min^–1^, and an Agilent HP-5MS column (60 m × 250 μm × 0.25 μm) was used for the chromatographic separation.

The temperature program started with an initial temperature of 75 °C. The temperature was increased by 15 °C min^–1^ up to 200 °C, then by 10 °C min^–1^ up to 280 °C. A final temperature ramp of 5 °C min^–1^ was applied up to 310 °C. This temperature was maintained for 10 min. A post-run phase was carried out at 310 °C for 20 min.

The mass spectrometer was operated in SIM mode, with a source temperature of 280 °C and a quadrupole temperature of 150 °C. Further details about the monitoring ions are reported in the SOP 02.^42^

A series of parameters were calculated for validating the method, including the method limit of detection and quantification (MDL and MQL, respectively), the linearity range, the sensitivity, the correlation coefficient (*R* ^2^), the trueness and bias, the matrix effect (*ME*%), and the repeatability (further details about the calculated parameters are reported in the SOP 02 ^42^). Procedural blanks were included in the analytical batch to monitor potential contamination and ensure robustness of the method.

To compare the performance of the different exploratory tests, trueness and bias were primarily used and were quantified by using the matrix-matched response factor. The same quantification method was applied for all trials carried out for the optimization of the preanalytical phase, whereas the quantification method for sample analysis was selected based on the test results.

Trueness and repeatability were calculated on six replicates of bovine fat samples spiked with 50 ng of native analytes and 50 ng of labeled standards.

*ME*% was calculated by comparing the slope of calibration curves constructed in solution (*m*_CS_) and in matrix (1 g of bovine adipose tissue) (*m*_CM_) according to the following formula

$$ME\%=\left(m_{CM}-m_{CS}\right)/m_{CS}$$

Seven concentration points, corresponding to analyte amounts ranging from 0 to 500 ng (0, 10, 25, 50, 100, 250, 500 ng per gram of matrix), were prepared, adding a constant amount of internal standard (50 ng). Concentration points outside the linearity range were excluded from the calibration curve. The graphs of the calibration curves are reported in the Supporting Information (Figure S1).

MDL and MQL values were calculated from the analysis of six procedural blanks subjected to the established preparative procedure, as the average of the blanks plus three times and ten times the standard deviation of the blank, respectively.

### 2.7 Tests on Real Human Adipose Tissue Sample

For the tests on human adipose tissue, the optimized preparative procedure was applied, as emerged from the results of all the combination tests (Figure 4). Quantification was carried out by using ^13^C-labeled PAH standards. The selection of the analytes to be determined in the samples was based on the results of method validation.

## 3 Results and Discussion

### 3.1 Choice and Optimization of the QuEChERS Procedures

#### 3.1.1 Exploratory Tests for the Determination of PAHs

Figure 1 shows the results of the trueness obtained from the exploratory tests. T2 and T8 deviated significantly from the 100% trueness (average bias of 118% and 119% for T2 and T8, respectively, Table S1). Additionally, applying the procedure T2, only 11 targeted congeners were identified out of 16, due to high background noise, which hindered the determination of the labeled standard. Tests showing a bias value between 30 and 50% were T3 and T7 (41 and 38%, respectively). Notably, for T3, all analytes were identified within a trueness of below 130%. In T1, T5, and T6, average bias values were between 20 and 30% (23, 22, and 24%, respectively). The test with the smallest deviation from 100% of trueness was T4 with a value of 19%. The only tests that allowed the identification of all 16 PAHs within the trueness range of 70–130% were T4 and T5, both performed using EtAc as solvent. Details of the trueness results for the different congeners are reported in the Supporting Information (Table S1).

**Figure 1:**
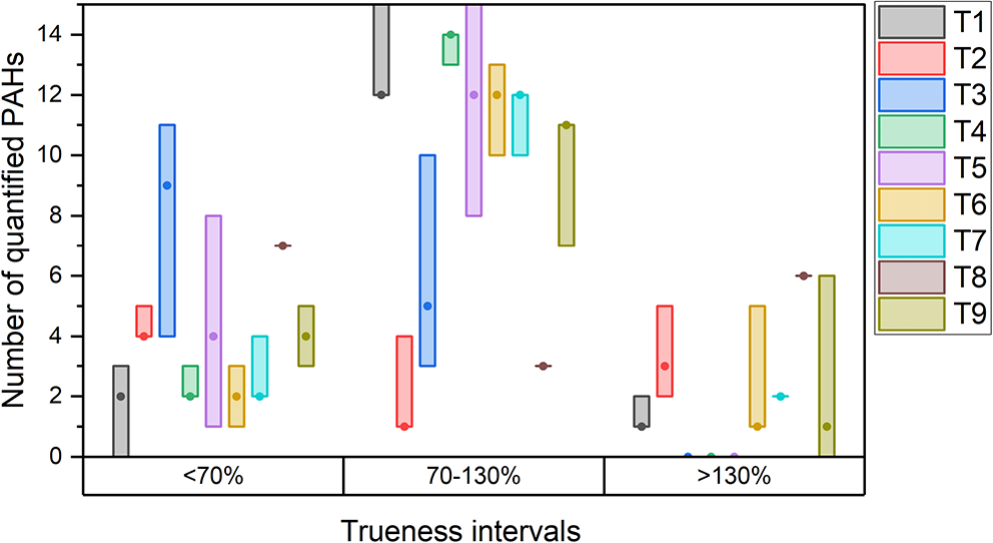
Distribution of the number of Polycyclic Aromatic Hydrocarbon (PAH) congeners quantified across analytical methods and trueness ranges. Box plots represent the full range (minimum to maximum) of the congener counts obtained from the three replicates. The circle within each box denotes the median value.

An important observation that enables an initial selection of parameters for the preanalytical phase concerns the presence of white solid residues in tests conducted with extraction solvents such as EtAc and Mix. These residues were not visible just after sample treatment and accumulated over time in the GC vials.

According to the literature, acetonitrile is the most commonly used solvent for extractions with the QuEChERS method and shows a high recovery of PAHs in fatty samples,^7^ with low-fat content extraction.^43^ In light of this, acetonitrile was chosen as the extraction solvent for this preparation.

Test results were analyzed using the Taguchi method to identify the optimal extraction salts, purification material, and quantity. For each variable, the average performance across all tests in which a specific option was used was calculated and compared with the averages of alternative options, as prescribed by the Taguchi approach. More details about the application of the Taguchi method for analytical method development with examples are provided in the SOP 04.^35^

A comparison of the average bias is shown in Figure 2.

**Figure 2:**
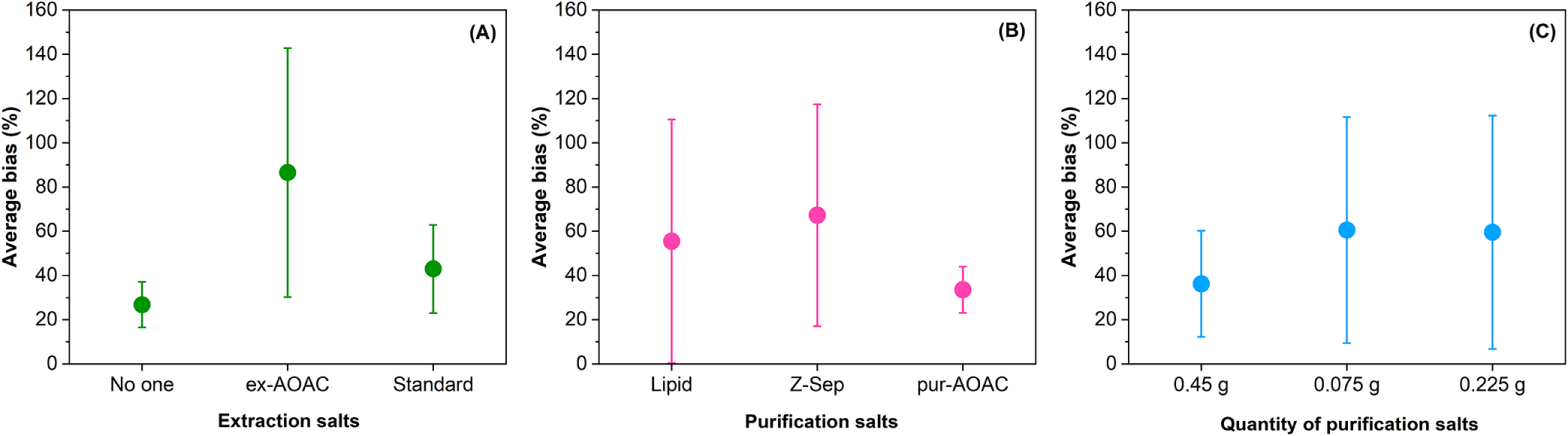
Comparison of parameters using the Taguchi method: (A) Extraction salts, (B) Purification salts, and (C) Quantity of purification salts. The dots are the average trueness deviations across all tests in which that specific option was used, and the brackets represent the standard deviation across the tests.

The best results for the amount of purifying agent were obtained with 0.450 g, while no significant differences in deviation values were noted between using 0.075 and 0.225 g (Figure 2C). Choosing a larger amount of purifying agent would also help reduce the amount of matrix that could enter the instrument, damage the column, and/or contaminate the source and quadrupole. Consequently, 0.450 g was selected as the amount of purifying agent.

Even if the variability across averaged tests is very high, the AOAC extraction salt option and the Z-Sep purifying agent were discarded due to higher average bias (Figure 2A,B), and the two other options for each parameter were retained for subsequent tests.

#### 3.1.2 Confirmation Tests

Additional tests were carried out to draw final considerations about the optimized procedure.

The effect of adding a concentration step on the method trueness was evaluated by comparing the same procedure (T10, ACN solvent, no extraction salts, 0.450 g of pur-AOAC as purification agent) with and without this additional step. We observed no increase in the bias caused by adding the concentration step to T10.

The results obtained from tests T10 and T11 were compared, as they were performed using the same procedure but differed in the use of extraction salts. T11 showed a lower deviation from 100% trueness compared to test T10 (41 vs 72% of average trueness).

Finally, from the comparison between the previous tests and T1, which differs from T10 only for the purification salts used (EMR-Lipid instead of pur-AOAC), we observed that T1 was the one performing best (average bias of 23%). The combination and results are also reported in the Supporting Information (Table S2).

Consequently, the preparative procedure used for T1 was chosen for testing human adipose tissue samples.

### 3.2 Method Validation

For validation of the analytical method, several parameters were evaluated in accordance with Section 2.6. The figures of merit evaluated include *ME*%, linearity range, sensitivity, *R* ^2^, trueness, repeatability, MDL, and MQL. The results obtained are summarized in Table 3.

**Table 3:** Parameters Used for the Method Validation

**PAHs**	***ME*%**	**linearity range**(ng g^**–1**^ **)**	**sensitivity (counts**g ng^**–1**^ **)**	***R*** ^**2**^	**trueness**(%)	**repeatability**(%)	**MDL**(ng g^**–1**^ **)**	**MQL**(ng g^**–1**^ **)**
**NA**	28	MDL-250	0.0239	0.9987	34	36	2.79	9.31
**ACL**	40	MDL-250	0.0287	0.9964	49	21	1.00	3.35
**AC**	47	MDL-250	0.0180	0.9917	60	18	4.13	13.76
**FL**	61	MDL-100	0.0216	0.9985	60	14	0.94	3.14
**PHE**	23	MDL-250	0.0222	0.9970	69	18	1.49	4.97
**AN**	22	MDL-250	0.0206	0.9998	66	13	0.20	0.66
**FA**	106	MDL-100	0.0419	0.9983	53	11	0.30	1.01
**Y**	60	MDL-250	0.0335	0.9964	55	14	0.31	1.02
**BaA**	121	MDL-250	0.0347	0.9929	69	19	0.11	0.38
**CHR**	106	MDL-250	0.0315	0.9906	75	17	0.13	0.44
**BbF**	10	MDL-250	0.0249	0.9930	78	14	0.35	1.17
**BkF**	13	MDL-250	0.0243	0.9950	73	13	0.34	1.15
**BaP**	15	MDL-250	0.0234	0.9994	72	11	0.33	1.09
**BgP**	26	MDL-250	0.0308	0.9952	64	13	0.30	1.01
**IcP**	30	MDL-250	0.0269	0.9979	64	11	0.31	1.05
**DhP**	26	MDL-250	0.0240	0.9835	68	17	0.35	1.16

The evaluation of *ME*% is important to determine whether the presence of such a complex matrix could influence the analyte’s response.

The comparison of the matrix-match calibration curve with the classical calibration curve in solution showed an appreciable difference in slope for most of the investigated analytes. In most cases, a *ME*% value greater than 20% is observed (Table 3). This indicates that the matrix has an impact on the analytical signal that cannot be ignored. Consequently, the matrix-match calibration curve was chosen as the quantification technique.

As reported in Table 3, the linearity range was MDL-250 ng g^–1^ for all congeners, except for FL and FA (MDL-100 ng g^–1^). Sensitivity values ranged from 0.02 to 0.04 counts g ng^–1^. All curves showed a high linearity, with *R* ^2^ values of all greater than 0.99, except for DhP.

As an example, Figure 3 reports the calibration curves for AN, which showed a matrix-matched calibration curve (CM) with equation *y* = 0.026*x* + 0.0231 and a solvent-based calibration curve (CS) with equation of *y* = 0.0169*x* – 0.995. The CM exhibited the highest *R* ^2^ among all the analyzed PAHs (*R* ^2^ = 0.9998, Table 3), indicating excellent linearity. For further details and to consult the calibration curves of the other analytes, please refer to Figure S1 of Supporting Information.

**Figure 3:**
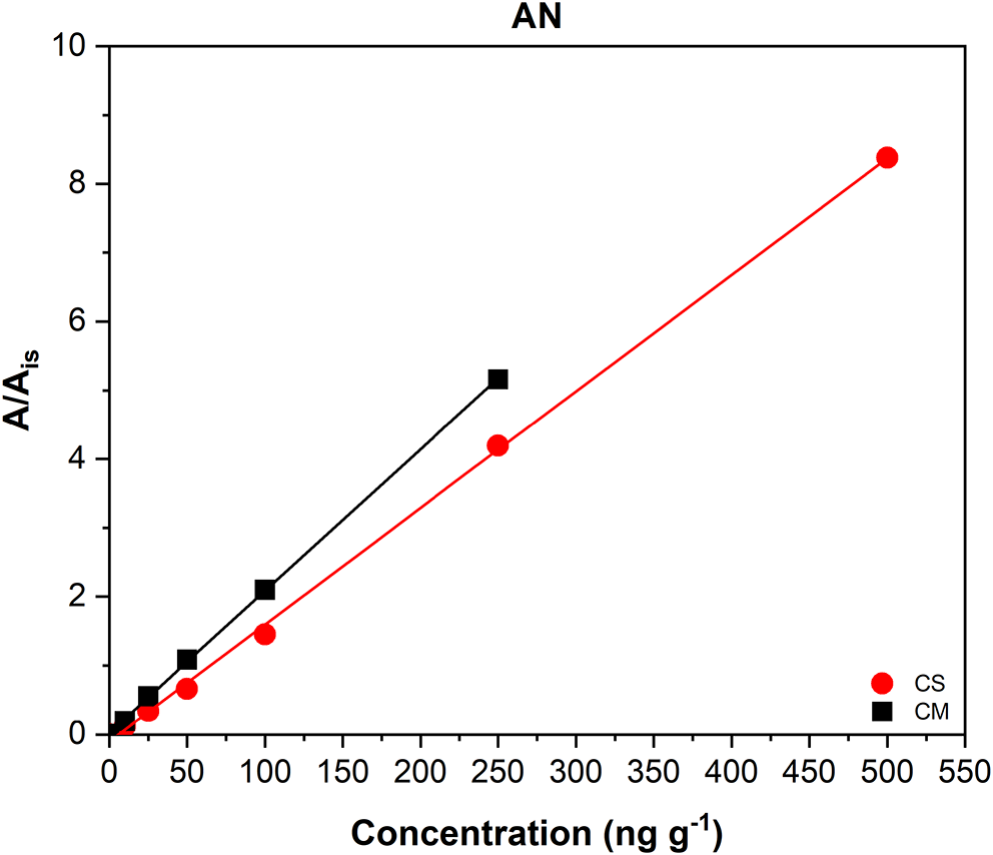
Calibration curve of AN in solution (CS) and in matrix (CM).

MDL values range from 0.11 to 4.13 ng g^–1^, while MQL values range from 0.38 to 13.76 ng g^–1^. Specifically, the highest MDL and MQL values were found for AC, while the lowest values were observed for BaA (Table 3). In the article by Sousa et al.^12^ in the literature, MDL values range from 0.04 to 12 ng g^–1^, while MQL values range from 0.1 to 39 ng g^–1^. It can be observed that the values obtained in this study are in line with the ranges reported in the literature.

The analyte with the highest trueness value was BbF (78%). Conversely, the analytes with the lowest trueness were NA and ACL, with an accuracy of 34 and 49%, respectively. Most congeners showed RSD values below 20% (between 11 and 18%). The only precision values above 20% were found for NA and ACL, at 36 and 21%, respectively.

Adipose tissue is poorly studied; therefore, there are no specific guidelines to assist in evaluating the results obtained, nor can standard methods be referenced, as is the case for environmental matrices. In general, due to the complexity of the matrix, higher deviation values are expected compared to simpler matrices. Sousa et al., in a recent article,^12^ report trueness values for PAHs in human adipose tissue that they consider suboptimal (ranging from 18 to 113%) as they fall outside the 70–120% range. Despite the low trueness, they chose not to exclude these analytes a priori but defined conditions to determine whether quantification is acceptable: trueness between 30 and 140% and repeatability below 20%. They also defined the need to correct the final result based on the measured trueness.

In this study, the same strategy is adopted to assess the acceptability of quantification. NA and ACL fall within the acceptable range for trueness but show precision values of >20% for repeated measurements. Consequently, these analytes are excluded from quantification in real samples.

The observed performance, while validating the method for the most toxicologically relevant compounds for which robustness and reliability were demonstrated, concurrently highlights some limitations. The significantly lower trueness and repeatability observed for the lighter PAHs restrict the method’s versatility. This limitation is consistent with analytical challenges reported in the literature, where the inherent volatility of low-molecular-weight PAHs often leads to evaporative losses during sample preparation steps and to higher MDL and MQL values for these analytes^12^ compared to the heavier congeners. When the focus is specifically on the analysis of volatile compounds, other techniques, such as Solid Phase Microextraction (SPME), are often better suited.^44,45^ The suboptimal results are strictly connected to the complexity of the matrix, for which a significant matrix effect was highlighted in this work. The decision to employ a matrix-matched calibration curve successfully mitigated this effect, but the magnitude of the ME can be an indicator of the challenges for achieving high accuracy in this specific matrix. To enhance the overall accuracy and repeatability, particularly for the lighter compounds, future work could focus on additional optimization of the extraction or purification procedures. Possible improvements include: (i) testing alternative cleanup sorbents to further reduce matrix interference or (ii) exploring modifications to the concentration step to minimize evaporative losses of volatile compounds. An option for enhancing the versatility and the number of potential PAHs quantifiable could be the adoption of alternative extraction techniques, known to perform better for volatile compounds, implemented as a separate, complementary protocol. This latter seems not convenient, since the lighter PAHs are less significant in the context of risk assessment for this specific matrix, the effort should be prioritized on optimizing the method for the high-molecular-weight PAHs, which are the main compounds of toxicological concern for human adipose tissue. Regarding technological improvements, the use of GC-MS/MS, often employed for complex matrices, could reduce the matrix background noise, allowing for better quantification even at low concentrations.

Furthermore, the reliance on bovine adipose tissue as a surrogate matrix for the validation phase represents a methodological limitation that should be acknowledged. Although minor interspecies differences in connective tissue density or microconstituent profiles may exist, the high degree of biochemical similarity between bovine and human fat^32^ supports the robustness of this approach. The performance of the EMR-Lipid cleanup is specifically optimized for linear hydrocarbon fatty acids.^46^ Since these constituents represent the bulk of the lipid mass in both bovine and human matrices, it is reasonable that the sorbent’s efficiency in removing matrix interferences remained consistent across both species, independent of the saturation. This strategic choice was necessitated by the scarcity of available samples and the invasive nature of human adipose tissue procurement, allowing the entire set of available human specimens (10 samples of 1–2 g each) to be reserved for final experimental analysis. Despite these supporting arguments, the potential for minor species-specific discrepancies cannot be fully ruled out and may contribute to the analytical uncertainty. Therefore, caution is warranted when interpreting the quantification data from human specimens. Further investigations involving a larger quantity of human material would be beneficial to definitively verify the consistency between these two matrices and confirm the absence of subtle species-specific matrix effects.

### 3.3 Tests on Real Human Adipose Tissue Sample

The treatment of human adipose tissue samples was carried out by applying the T1 procedure with target analytes AC, FL, PHE, AN, FA, Y, BaA, CHR, BbF, BkF, BaP, BgP, IcP, and DhP, quantified by applying the matrix-match calibration curve previously prepared in a surrogate matrix. The procedure pipeline is summarized in Figure 4.

**Figure 4:**
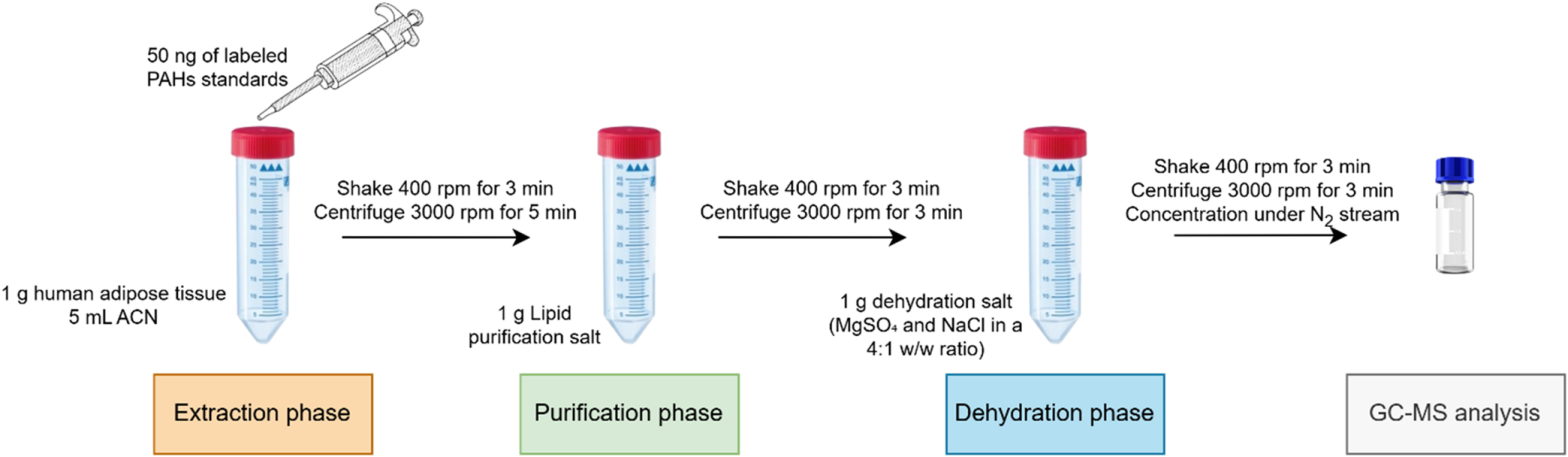
Procedure used for the test with human adipose tissue samples.

Assuming that the sensitivity was the same inside and outside the linearity range, the concentration of analytes in the samples was calculated based on this sensitivity, along with MDL and MQL.

In the human samples assayed, the presence of three analytes was confirmed: PHE, AN, and Y. PHE was detected in four different samples (#2, #5, #6, and #10). However, in the case of samples #2 and #10, it was not detected in either duplicate. AN was detected in three samples (nos. 2, 5, and #10), in at least one of the replicates. Y was detected in 9 out of 10 analyzed samples (not detected in sample #7) and was the only PAH for which quantification was possible in some samples. In samples #2, #4, #5, #6, and #9, it was quantified at concentrations ranging from 2.34 to 4.88 ng g^–1^. The complete results obtained for human adipose tissues are reported in the Supporting Information (Table S3).

Among the analyzed compounds, only Y showed concentrations above the MQL. The concentrations observed in our study were notably higher than those reported in the literature, where levels were generally lower (lower than 0.252 ng g^–1^ ^18^) or below the MQL,^12^ suggesting a potential risk of exposure or bioaccumulation of pyrene in these patients.

Conversely, other studies^12,18^ detected a series of low-molecular-weight congeners that were not observed in our sample set. It should be noted that both the MDL and MQL were higher for low-molecular-weight compounds compared to heavier ones, reaching their highest values for AC (4.13 ng g^–1^) and NA (2.79 ng g^–1^), which were therefore excluded from quantification in this work. It is thus possible that, although this method performs well for most PAHs, it may tend to underestimate or fail to detect lighter compounds. Sousa et al.^12^ also reported higher MDL and MQL values for naphthalene and acenaphthylene, compared to higher-molecular-weight PAHs, suggesting that these compounds often present a greater analytical challenge. However, this focus on higher-molecular-weight PAHs is consistent with risk assessment goals, as these congeners typically possess greater carcinogenic potency and thus are more relevant for long-term bioaccumulation in human adipose tissue.

## 4 Conclusions

The results of the present study highlight that the adoption of the procedure based on the QuEChERS method, integrated with GC-single quadrupole MS analysis, represents a valid approach for the quantitative determination of 14 PAHs in human adipose tissue.

Due to its complexity and the invasiveness of sampling, human adipose tissue remains scarcely investigated, with no reference methods currently available for PAHs analysis. However, given the toxicity and lipophilicity of these compounds, targeted studies are essential to advance biomonitoring research. Among the 16 congeners tested, the observed significant matrix effects restricted the method’s applicability to 14 PAHs, since naphthalene and acenaphthylene, the most low-molecular-weight and volatile congeners, are not efficiently quantifiable. Thus, we propose our method as an effective option for the quantitative determination of the more toxic, high-molecular-weight PAHs, which are of greater concern for human health. The proposed method is one of the few able to detect these compounds in such a complex matrix and, to the best of our knowledge, the only one that uses the QuEChERS method combined with GC-MS. This innovative approach, being the first to integrate these two techniques for the determination of PAHs in adipose tissue, offers the potential to standardize a protocol that combines speed, greenness, and reduced costs, maintaining a high reliability of the results, due to the high sensitivity and selectivity of the GC-MS analytical technique.

The analysis of ten human adipose tissue samples confirms the presence of phenanthrene, anthracene, and pyrene, which were detected in several samples. Despite the small sample size does not allow for generalizing conclusions about the prevalence of PAHs in the population, these results indicate a potential risk of environmental exposure to these substances and reinforce the need for biomonitoring studies. Further studies on the bioaccumulation of these substances in healthy individuals and patients with diseases are needed.

This research demonstrates substantial progress in the development of an innovative analytical procedure. However, it highlights areas for improvement, particularly in terms of trueness and repeatability: additional optimization of the extraction or purification procedures (i.e., testing new purification material) may help enhance the accurate quantification of lighter analytes. Also, the employment of different analytical techniques, such as tandem GC-MS, could help in reducing the matrix effect and obtaining better results. In addition to aspects related to analytical chemistry, future studies could involve comparing the levels of PAHs in different types of human fat or in various organs.

Moreover, the proposed method could be applied to animal adipose tissue samples to conduct bioaccumulation and ecotoxicity studies. Furthermore, the preanalytical method could serve as the basis for developing procedures to extract other priority or emerging pollutants from adipose matrices, in the context of both biomonitoring and ecotoxicity studies. In an ecological context, the investigation of the bioaccumulation processes of lipophilic pollutants in polar fauna, which is particularly vulnerable to global contamination phenomena, could be an interesting avenue for future research.

## Supplementary Material



## Notes

Details about experimental conditions and results obtained during the method optimization from exploratory tests; matrix-match calibration curves and solution-based curves; concentration data of PAHs on human samples (PDF)

## References

[1] Sousa S., Maia M. L., Delerue-Matos C., Calhau C., Domingues V. F. (2022). The Role
of Adipose Tissue Analysis on Environmental Pollutants Biomonitoring
in Women: The European Scenario. Sci. Total
Environ..

[2] Aslam R., Sharif F., Baqar M., Shahzad L. (2022). Source Identification
and Risk Assessment of Polycyclic Aromatic Hydrocarbons (PAHs) in
Air and Dust Samples of Lahore City. Sci. Rep..

[3] Sampaio G. R., Guizellini G. M., Da Silva S. A., De Almeida A. P., Pinaffi-Langley A. C. C., Rogero M. M., De Camargo A. C., Torres E. A. F. S. (2021). Polycyclic Aromatic Hydrocarbons in Foods: Biological
Effects, Legislation, Occurrence, Analytical Methods, and Strategies
to Reduce Their Formation. IJMS.

[4] Yu G. W., Laseter J., Mylander C. (2011). Persistent
Organic Pollutants in
Serum and Several Different Fat Compartments in Humans. J. Environ. Public Health.

[5] Ciganek M., Neca J. (2006). Polycyclic Aromatic Hydrocarbons in Porcine and Bovine Organs and
Tissues. Vet. Med..

[6] Jin X., Hua Q., Liu Y., Wu Z., Xu D., Ren Q., Zhao W., Guo X. (2021). Organ and
Tissue-Specific Distribution
of Selected Polycyclic Aromatic Hydrocarbons (PAHs) in ApoE-KO Mouse. Environ. Pollut..

[7] Lucas, D. ; Zhao, L. PAH Analysis in Salmon with Enhanced Matrix Removal, Application Note; Agilent Technologies, Inc.: Santa Clara, CA, USA, 2015.

[8] Forsberg N. D., Wilson G. R., Anderson K. A. (2011). Determination of Parent and Substituted
Polycyclic Aromatic Hydrocarbons in High-Fat Salmon Using a Modified
QuEChERS Extraction, Dispersive SPE and GC–MS. J. Agric. Food Chem..

[9] Mercogliano R., Santonicola S., De Felice A., Anastasio A., Murru N., Ferrante M. C., Cortesi M. L. (2016). Occurrence and Distribution
of Polycyclic Aromatic Hydrocarbons in Mussels from the Gulf of Naples,
Tyrrhenian Sea, Italy. Mar. Pollut. Bull..

[10] Serpe F. P., Esposito M., Gallo P., Serpe L. (2010). Optimisation and Validation
of an HPLC Method for Determination of Polycyclic Aromatic Hydrocarbons
(PAHs) in Mussels. Food Chem..

[11] Lund M., Duedahl-Olesen L., Christensen J. H. (2009). Extraction of Polycyclic Aromatic
Hydrocarbons from Smoked Fish Using Pressurized Liquid Extraction
with Integrated Fat Removal. Talanta.

[12] Sousa S., Paíga P., Pestana D., Faria G., Delerue-Matos C., João Ramalhosa M., Calhau C., Fernandes
Domingues V. (2023). Optimization of a Simple, Effective, and Greener Methodology
for Polycyclic Aromatic Hydrocarbon Extraction from Human Adipose
Tissue. Anal. Methods.

[13] Sousa S., Paíga P., Pestana D., Faria G., Delerue-Matos C., Ramalhosa M. J., Calhau C., Domingues V. F. (2024). Evaluating
the Impact of Polycyclic Aromatic Hydrocarbon Bioaccumulation in Adipose
Tissue of Obese Women. Chemosphere.

[14] Vorkamp K., Esteban López M., Gilles L., Göen T., Govarts E., Hajeb P., Katsonouri A., Knudsen L. E., Kolossa-Gehring M., Lindh C., Nübler S., Pedraza-Díaz S., Santonen T., Castaño A. (2023). Coordination
of Chemical Analyses under the European Human Biomonitoring Initiative
(HBM4 EU): Concepts, Procedures and Lessons Learnt. Int. J. Hyg. Environ. Health.

[15] Yang Z., Guo C., Li Q., Zhong Y., Ma S., Zhou J., Li X., Huang R., Yu Y. (2021). Human Health Risks Estimations from
Polycyclic Aromatic Hydrocarbons in Serum and Their Hydroxylated Metabolites
in Paired Urine Samples. Environ. Pollut..

[16] Zhang S., Luo W., Zhao F., Huang L., Qin R., Yan X., Tang B., Luo X., Mai B., Yu Y., Zheng J. (2024). Melanin-Mediated Accumulation
of Polycyclic Aromatic Hydrocarbons
in Human Hair: Insights from Biomonitoring and Cell Exposure Studies. J. Hazard. Mater..

[17] Lau E. V., Gan S., Ng H. K. (2010). Extraction Techniques
for Polycyclic Aromatic Hydrocarbons
in Soils. Int. J. Anal. Chem..

[18] Pastor-Belda M., Campillo N., Arroyo-Manzanares N., Torres C., Pérez-Cárceles M. D., Hernández-Córdoba M., Viñas P. (2019). Bioaccumulation
of Polycyclic Aromatic Hydrocarbons for Forensic Assessment Using
Gas Chromatography–Mass Spectrometry. Chem. Res. Toxicol..

[19] Moon H.-B., Lee D.-H., Lee Y. S., Kannan K. (2012). Occurrence and Accumulation
Patterns of Polycyclic Aromatic Hydrocarbons and Synthetic Musk Compounds
in Adipose Tissues of Korean Females. Chemosphere.

[20] Perestrelo R., Silva P., Porto-Figueira P., Pereira J. A. M., Silva C., Medina S., Câmara J. S. (2019). QuEChERS - Fundamentals, Relevant
Improvements, Applications and Future Trends. Anal. Chim. Acta.

[21] Rouvière F., Buleté A., Cren-Olivé C., Arnaudguilhem C. (2012). Multiresidue
Analysis of Aromatic Organochlorines in Soil by Gas Chromatography-Mass
Spectrometry and QuEChERS Extraction Based on Water/Dichloromethane
Partitioning. Comparison with Accelerated Solvent Extraction. Talanta.

[22] Anastassiades M., Lehotay S. J., Štajnbaher D., Schenck F. J. (2003). Fast and Easy Multiresidue
Method Employing Acetonitrile Extraction/Partitioning and “Dispersive
Solid-Phase Extraction” for the Determination of Pesticide
Residues in Produce. J. AOAC Int..

[23] Kiełbasa A., Buszewski B. (2017). PAHs in Animal
Tissues – the Analytics of PAHs
in New Reference Materials and Their Homogeneity. Anal. Methods.

[24] Townsend R., van Keulen G., Desbrow C., Godfrey A. R. (2020). An investigation
of the utility of QuEChERS for extracting acid, base, neutral and
amphiphilic species from example environmental and clinical matrices. Anal. Sci. Adv..

[25] Nisbet I. C. T., LaGoy P. K. (1992). Toxic Equivalency
Factors (TEFs) for Polycyclic Aromatic
Hydrocarbons (PAHs). Regul. Toxicol. Pharmacol..

[26] Food and Drug Administration (FDA) . M10 Bioanalytical method validation and study sample analysis - Guidance for Industry. Report n. FDA-2019-D-1469, 2022.

[27] Siemiątkowska A., Wassef A., Sadek R., Park C., Yohn C., Brunetti L., Kagan L. (2022). A validated LC-MS/MS method for the
quantitation of cefazolin in human adipose tissue: Application of
EMR-Lipid sorbent as an efficient sample clean-up before mass spectrometric
analyses. A validated LC-MS/MS method for the quantitation of cefazolin
in human adipose tissue: Application of EMR-Lipid sorbent as an efficient
sample clean-up before mass spectrometric analyses. J. Pharm. Biomed. Anal..

[28] Noll B. D., Coller J. K., Somogyi A. A., Morris R. G., Russ G. R., Hesselink D. A., Van Gelder T., Sallustio B. C. (2013). Validation
of an LC–MS/MS Method to measure tacrolimus in rat kidney and
liver tissue and its application to human kidney biopsies. Ther. Drug Monit..

[29] Roseboom I. C., Thijssen B., Rosing H., Alves A., Younis B. M., Musa A. M., Beijnen J. S., Dorlo T. P. C. (2022). Development
and
validation of an ultra-high performance liquid chromatography coupled
to tandem mass spectrometry method for the quantification of the antileishmanial
drug paromomycin in human skin tissue. J. Chromatogr.
B.

[30] Jin Y., Song Z., Du H., Zhuo J., Zhang X., Wang Y., Xu H., Lu X. (2025). Quantification of Trans-
and Cis-Vitamin K1 Isomers in Human Plasma by a Rapid and Sensitive
LC–MS/MS Method With Surrogate Matrix. Biomed. Chromatogr..

[31] De
Palma R., Matta M., Florian J., Patel V., Rouse R. (2025). Absolute quantitation of neopterin as an endogenous pharmacodynamic
Biomarker: The successful method development, validation, and use
of a surrogate matrix for clinical sample analysis. Anal. Biochem..

[32] Gurr, M. I. ; Harwood, J. L. ; Frayn, K. N. ; Murphy, D. J. ; Michell, R. H. Lipids: Biochemistry, Biotechnology and Health, 6th ed.; Wiley-Blackwell, 2016.

[33] Stenerson, K. K. Extraction and Analysis of PAHs from Grilled Hamburger Using Supel QuE Z-Sep QuEChERS Sorbents and SPB-608 Capillary Columns, Reporter 32.1; Sigma-Aldrich (Supelco): Bellefonte, PA, 2014.

[34] Krishnaiah, K. ; Shahabudeen, P. Applied Design of Experiment and Taguchi Methods; PHI Learning Private Limited: New Delhi, 2012.

[35] Gregoris, E. ; Franchin, A. ; Roman, M. SOP 04 Setting up an Experimental Design Using the Taguchi Approach for Bioanalytical Method Development. 2025 10.71731/DATA_UNIVE/4PVYYJ.

[36] How to Use QuEChERS for Diverse Sample Types 2025 https://www.restek.com/global/it/articles/how-to-use-quechers-for-diverse-sample-types?srsltid=AfmBOoq0BNT0jnkE7pGvCezWDnIzqiANBtEq4V-x5KAlT7NgK51Kaksu&utm_source=chatgpt.com (accessed March 05, ).

[37] Łozowicka B., Rutkowska E., Jankowska M. (2017). Influence of QuEChERS Modifications
on Recovery and Matrix Effect during the Multi-Residue Pesticide Analysis
in Soil by GC/MS/MS and GC/ECD/NPD. Environ.
Sci. Pollut Res. Int..

[38] Cajka T., Sandy C., Bachanova V., Drabova L., Kalachova K., Pulkrabova J., Hajslova J. (2012). Streamlining Sample Preparation and
Gas Chromatography–Tandem Mass Spectrometry Analysis of Multiple
Pesticide Residues in Tea. Anal. Chim. Acta.

[39] Khan, A. Analysis of 18 Polycyclic Aromatic Hydrocarbons in Soil Using the QuEChERS Method, Application Note 20677; Thermo Fisher Scientific: Runcorn, Cheshire, UK, 2014.

[40] Sadowska-Rociek A., Surma M., Cieślik E. (2014). Comparison
of Different Modifications
on QuEChERS Sample Preparation Method for PAHs Determination in Black,
Green, Red and White Tea. Environ. Sci. Pollut
Res..

[41] Design of Experiments in Production Engineering. In Management and Industrial Engineering; Davim, J. P. , Ed.; Springer International Publishing: Cham, 2016 10.1007/978-3-319-23838-8.

[42] Gregoris, E. ; Franchin, A. ; Sorarù, L. ; Roman, M. SOP 02 Analysis of Polycyclic Aromatic Hydrocarbons in Adipose Tissue by GC-MS. 2025 10.71731/DATA_UNIVE/WSQSCS. PMC12980236 41835556

[43] Cloutier P.-L., Fortin F., Groleau P. E., Brousseau P., Fournier M., Desrosiers M. (2017). QuEChERS Extraction for Multi-Residue
Analysis of PCBs, PAHs, PBDEs and PCDD/Fs in Biological Samples. Talanta.

[44] Menezes H. C., Paulo B. P., Costa N. T., Cardeal Z. L. (2013). New method
to determination
of naphthalene in ambient air using cold fiber-solid phase microextraction
and gas chromatography–mass spectrometry. Microchem. J..

[45] Watanabe K., Fujita H., Hasegawa K., Gonmori K., Suzuki O. (2011). GC/MS with
Post-Column Switching for Large Volume Injection of Headspace Samples:
Sensitive Determination of Volatile Organic Compounds in Human Whole
Blood and Urine. Anal. Chem..

[46] Apffel A., Zhao L., Sartain M. J. (2021). A Novel
Solid Phase Extraction Sample
Preparation Method for Lipidomic Analysis of Human Plasma Using Liquid
Chromatography/Mass Spectrometry. Metabolites.

