# Supplementary material for: First Application of QuEChERS-GC-MS Analysis for Polycyclic Aromatic Hydrocarbons Detection in Human Adipose Tissue
Source: ACS Omega. 2026 Feb 25;11(9):14287–95. doi: 10.1021/acsomega.5c06719 (PMC12980236; doi:10.1021/acsomega.5c06719)
Supplement: Supplementary file 1 [file ao5c06719_si_001.pdf]

SUPPORTING INFORMATION

ADDITIONAL EXPERIMENT DETAILS AND RESULTS

Of the paper

First Application of QuEChERS-GC-MS Analysis for Polycyclic Aromatic Hydrocarbons Detection in Human Adipose Tissue

Alice Franchin[a], Elena Gregoris[b,*], Luca Sorarù[a], Elena Stocco[c], Vincenzo Vindigni[c], Andrea Porzionato[c], Daniele Brunelli[c], Veronica Macchi[c], Andrea Gambaro[a], Marco Roman[a]

[a] Department of Environmental Sciences, Informatic and statistics, Ca' Foscari University of Venice, via Torino 155, 30172, Venice, Italy
[b] Institute of Polar Sciences, National Research Council (ISP-CNR), via Torino 155, 30172, Venice, Italy
[c] Department of Neurosciences, University of Padua, via Belzoni 160, 35121, Padua, Italy

* Corresponding author: elena.gregoris@cnr.it

METHOD OPTIMISATION

This section of the Supplementary Information reports additional details concerning the experimental conditions or results obtained during the method optimisation, that were not included in the main text of the article.

The following table (Table S1) reports the average trueness percentages, calculated across the three replicates, obtained from the exploratory tests selected by using the Taguchi method (T1–T9). For each test, the total number of quantified PAHs and the average deviation were calculated to evaluate the overall performance and reproducibility of the extraction and purification conditions.

Table S1 – Trueness, number of quantified PAHs and average bias values for each tests

PAHs	T1	T2	T3	T4	T5	T6	T7	T8	T9
**NA**	178%	82%	94%	72%	74%	76%	89%	27%	57%
**ACL**	95%	-	84%	82%	78%	100%	98%	65%	85%
**AC**	85%	-	42%	74%	77%	78%	321%	114%	41%
**FL**	113%	77%	71%	74%	72%	72%	225%	37%	80%
**PHE**	101%	584%	98%	73%	68%	91%	71%	36%	61%
**AN**	73%	80%	77%	81%	74%	113%	107%	68%	92%
**FA**	82%	474%	79%	102%	70%	91%	83%	49%	76%
**Y**	75%	290%	73%	80%	75%	92%	92%	66%	82%
**BaA**	81%	59%	0%	81%	65%	109%	85%	77%	81%
**CHR**	76%	82%	0%	85%	67%	172%	87%	76%	76%
**BbF**	78%	121%	42%	85%	95%	118%	86%	305%	222%
**BkF**	72%	143%	43%	90%	86%	135%	111%	184%	261%
**BaP**	75%	23%	54%	79%	76%	131%	83%	440%	113%
**BgP**	72%	-	74%	83%	89%	110%	94%	355%	269%
**IcP**	63%	-	58%	76%	103%	78%	41%	181%	235%
**DhP**	94%	-	59%	88%	106%	20%	41%	626%	253%
**Total quantified PAHs**	16	11	16	16	16	16	16	16	16
**Average deviation**	23%	118%	41%	19%	22%	24%	38%	119%	64%

The experimental conditions of additional tests, performed after the first exploratory tests planned by using the Taguchi method, are summarised in Table S2. The experimental conditions include solvent type, extraction salts, purification salts, and their quantities. Table S2 also reports whether a nitrogen concentration step was applied. For each experiment, the corresponding average deviation values, calculated across the three replicates, together with the variability range of such value (minimum and maximum) is reported.

Table S2 – Combinations of the parameters and results of the confirmation tests

Test	Solvent	Extraction salts	Purification salts	Quantity of purification salts	Concentration with nitrogen	Deviation values	Minimum deviation value	Maximum deviation value
**T1**	ACN	No one	Lipid	0.450 g	No	23%	1%	78%
**T10**	ACN	No one	pur-AOAC	0.450 g	No	72%	10%	251%
**T10C**	ACN	No one	pur-AOAC	0.450 g	Yes	39%	13%	56%
**T11**	ACN	Standard	pur-AOAC	0.450 g	No	41%	4%	100%

Figure S1 shows the matrix-match calibration curves and the solution-based calibration curves for the 16 target PAHs.

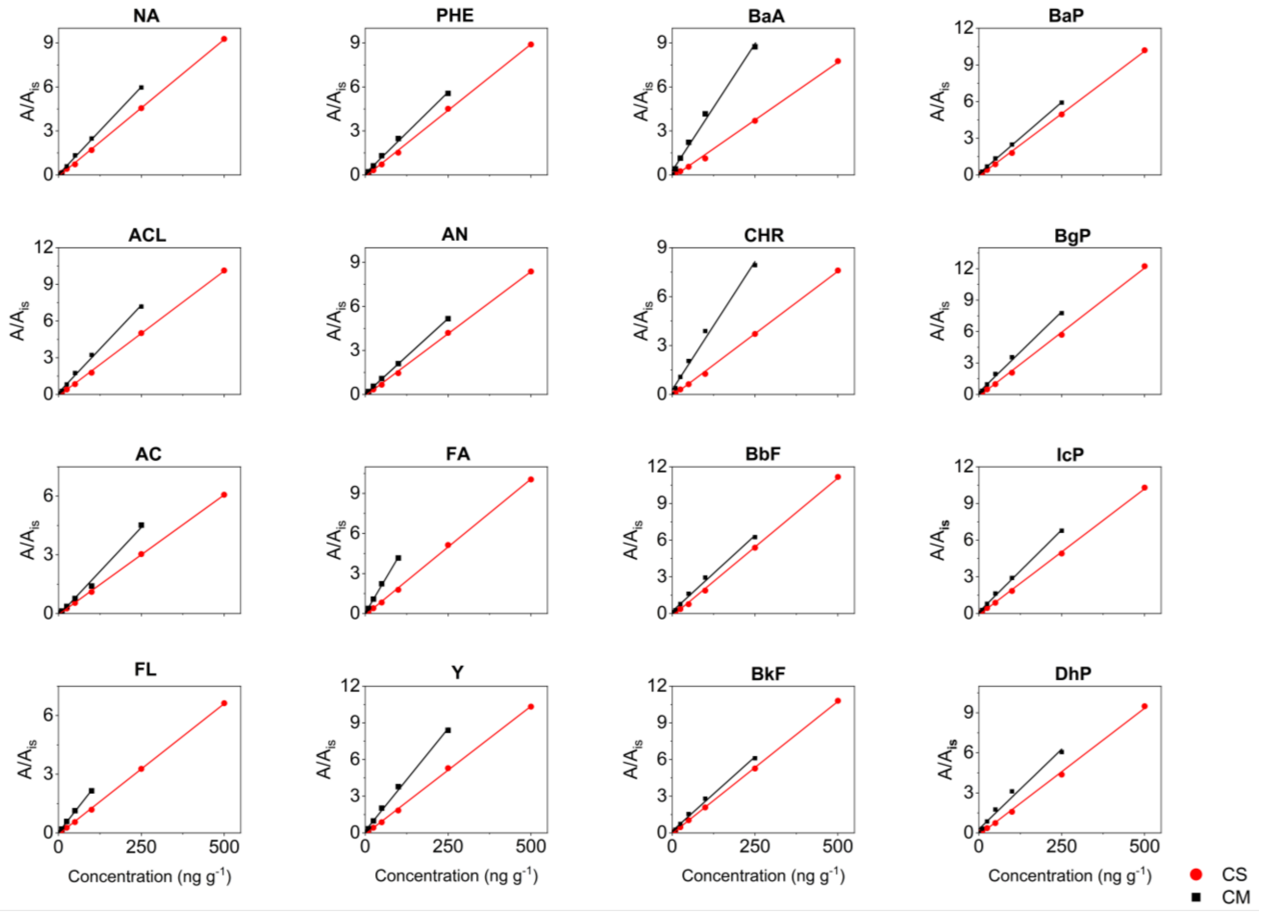

Figure S1 - Calibration curves constructed in solution (CS) and in matrix (CM)

RESULTS

This section reports the concentrations of the 14 PAHs determined in human adipose tissue samples from 10 donors (values in ng g^{-1}), by using the optimised method. In Table S3, "nd" indicates compounds not detected (values below the detection limit, MDL), and "DET" indicates compounds detected (above MDL) but below the method quantification limit (MQL). The table includes the replicate analyses (1 and 2) performed for each sample. The tissue site (arm, abdomen, or breast) is also reported.

Table S3 – PAHs detected and quantified in human adipose tissue samples. Values in ng g^{-1}. nd: non detected; DET: detected (non quantifiable).

		PAHs													
**Samples**	**Test**	**AC**	**FL**	**PHE**	**AN**	**FA**	**Y**	**BaA**	**CHR**	**BbF**	**BkF**	**BaP**	**BgP**	**IcP**	**DhP**
#1 (abdomen)	1	nd	nd	nd	nd	nd	DET	nd	Nd	nd	nd	nd	nd	nd	nd
	2	nd	nd	nd	nd	nd	DET	nd	Nd	nd	nd	nd	nd	nd	nd
#2 (arm)	1	nd	nd	DET	DET	nd	4.88	nd	Nd	nd	nd	nd	nd	nd	nd
	2	nd	nd	nd	DET	nd	4.81	nd	Nd	nd	nd	nd	nd	nd	nd
#3 (abdomen)	1	nd	nd	nd	nd	nd	DET	nd	Nd	nd	nd	nd	nd	nd	nd
	2	nd	nd	nd	nd	nd	DET	nd	Nd	nd	nd	nd	nd	nd	nd
#4 (abdomen)	1	nd	nd	nd	nd	nd	3.05	nd	Nd	nd	nd	nd	nd	nd	nd
#5 (arm)	1	nd	nd	DET	DET	nd	4.73	nd	Nd	nd	nd	nd	nd	nd	nd
	2	nd	nd	DET	nd	nd	3.20	nd	Nd	nd	nd	nd	nd	nd	nd
#6 (breast)	1	nd	nd	DET	nd	nd	3.61	nd	Nd	nd	nd	nd	nd	nd	nd
#7 (abdomen)	1	nd	nd	nd	nd	nd	nd	nd	Nd	nd	nd	nd	nd	nd	nd
	2	nd	nd	nd	nd	nd	nd	nd	Nd	nd	nd	nd	nd	nd	nd

		PAHs													
Samples	Test	AC	FL	PHE	AN	FA	Y	BaA	CHR	BbF	BkF	BaP	BgP	IcP	DhP
#8 (abdomen)	1	nd	nd	nd	nd	nd	2.34	nd	Nd	nd	nd	nd	nd	nd	nd
	2	nd	nd	nd	nd	nd	2.80	nd	Nd	nd	nd	nd	nd	nd	nd
#9 (abdomen)	1	nd	nd	nd	nd	nd	DET	nd	Nd	nd	nd	nd	nd	nd	nd
	2	nd	nd	nd	nd	nd	2.74	nd	Nd	nd	nd	nd	nd	nd	nd
#10 (abdomen)	1	nd	nd	nd	nd	nd	DET	nd	Nd	nd	nd	nd	nd	nd	nd
	2	nd	nd	DET	DET	nd	2.46	nd	Nd	nd	nd	nd	nd	nd	nd
